# Spectroscopic identification of Ca-bearing uranyl silicates formed in C–S–H systems

**DOI:** 10.1038/s41598-023-30024-0

**Published:** 2023-02-28

**Authors:** Antonia S. Yorkshire, Martin C. Stennett, Brant Walkley, John L. Provis, Luke T. Townsend, Latham T. Haigh, Neil C. Hyatt, Lucy M. Mottram, Claire L. Corkhill

**Affiliations:** 1 Immobilisation Science Laboratory, Department of Materials Science and Engineering, University of Sheffield, Sheffield, UK; 2 Department of Chemical and Biological Engineering, University of Sheffield, Sheffield, UK

**Keywords:** Nuclear waste, Nuclear chemistry

## Abstract

Portland cement-based grouts used for radioactive waste immobilisation contain a Ca- and Si-rich binder phase, known as calcium–silicate–hydrate (C–S–H). Depending on the blend of cement used, the Ca/Si ratio can vary considerably. A range of C–S–H minerals with Ca/Si ratios from 0.6 to 1.6 were synthesised and contacted with aqueous U(VI) at 0.5 mM and 10 mM concentrations. Solid-state ^29^Si MAS-NMR spectroscopy was applied to probe the Si coordination environment in U(VI)-contacted C–S–H minerals and, in conjunction with U L_III_-edge X-ray absorption spectroscopy analysis, inferences of the fate of U(VI) in these systems were made. At moderate or high Ca/Si ratios, uranophane-type uranyl silicates or Ca-uranates dominated, while at the lowest Ca/Si ratios, the formation of a Ca-bearing uranyl silicate mineral, similar to haiweeite (Ca[(UO_2_)_2_Si_5_O_12_(OH)_2_]·3H_2_O) or Ca-bearing weeksite (Ca_2_(UO_2_)_2_Si_6_O_15_·10H_2_O) was identified. This study highlights the influence of Ca/Si ratio on uranyl sequestration, of interest in the development of post-closure safety models for U-bearing radioactive waste disposal.

## Introduction

Cement materials are used widely throughout the nuclear industry, in construction and also in the direct stabilisation/solidification of radioactive wastes. Such materials will continue to be used extensively in radioactive waste management applications in the future and, particularly so, in the construction of cementitious geological disposal facilities.

The main component of the most commonly used cement material, Portland cement, is the calcium–silicate–hydrate (“C–S–H”) binder phase. This phase makes up at least ~ 50 wt% of a hardened Portland cement, but varies in its stoichiometry due to differences in Ca, Si and water content. For Portland cement-based grouts used within the nuclear industry, variations in the Ca and Si content (i.e., the Ca/Si molar ratio) are induced by siliceous or lime-based powders (e.g. Ca(OH)_2_, CaCO_3_), known as supplementary cementitious materials (SCMs), which are added to Portland cements to lower the heat output during hydration and improve the fluidity^1^. Examples of siliceous SCMs include blast-furnace slag (BFS), fly ash (FA) and silica fume (SF), which can be used to produce encapsulating grouts for the immobilisation of specific waste streams^2^. Cements and concretes containing SF may also be used to line and plug the vaults of geological disposal facilities^3^, with significant silica addition giving rise to a C–S–H phase with a low Ca content (i.e. a lower Ca/Si ratio)^3,4^. Addition of lime-based powders to Portland cement has been considered in the production of high-pH backfill cement for geological disposal facilities (i.e. Nirex Reference Vault Backfill; NRVB)^5^. In this scenario, the alkaline pH encourages sorption and binding of selective radionuclides, such as U and other actinides, and lowers their solubility^6,7^. The higher Ca content of such cements results in a C–S–H phase with a higher Ca/Si ratio.

The relative age of a cementitious based geological disposal facility will also dictate the Ca/Si ratio of C–S–H formed in construction or backfilling cements. It is expected that a fresher, “young” hydrating cement will form C–S–H phases that are rich in Ca. With time, as groundwater ingresses the facility, these C–S–H phases will become Ca-depleted due to Ca-leaching and carbonate ingress (i.e. carbonation of Ca in C–S–H phases)^8^.

Historical and current intermediate level waste (ILW) streams are known to contain U (and other actinides, including Pu, and U/Pu daughter nuclides)^9^ and, more recently, options considered for the management of large surplus remains of depleted U (i.e., ^238^UO_3_ and ^238^U_3_O_8_ powders arising from fuel reprocessing and fabrication operations) also include the potential use of cement materials^10^. For example, the baseline treatment option for depleted U in the UK is encapsulation within a cement grout, similar to the BFS:PC cement grout used for encapsulation of spent UO_2_ fuel cladding. Alternatively, it may be mixed with a concrete to form a Depleted Uranium Aggregate (DUAGG) that could be suitable to backfill vaults in a geological disposal facility^10,11^.

In all of the aforementioned scenarios, there is potential for U to come into contact with hydrated cement materials. Previous studies have already demonstrated that C–S–H phases show efficient U(VI) uptake and/or secondary U(VI) phase precipitation^12–15^. The U(VI) coordination environment in these studies has often been compared to that of uranyl silicate minerals, such as uranophane (Ca(UO_2_)_2_SiO_3_(OH)_2_·5H_2_O) or soddyite ((UO_2_)_2_SiO_4_·2H_2_O). In systems with a higher abundance of Ca (e.g. high Ca/Si ratios), the solubility of U(VI) is largely controlled by the formation of calcium uranate (CaUO_4_.*x*H_2_O) phases^16^. The effect of Ca/Si ratio of C–S–H on this uptake has not previously been considered in detail, nor have higher concentrations of U, relevant to the scenarios described above.

In this study, pure C–S–H phases with target Ca/Si ratios of 0.6, 0.8, 1.0, 1.2 and 1.6 were synthesised and contacted with aqueous U(VI) at both low and high concentration. The local chemistry and coordination of the secondary U(VI) phases formed in, or in conjunction with, the C–S–H minerals was probed using U L_III_-edge X-ray absorption spectroscopy (XAS). Characterisation of the structural modification induced in the C–S–H minerals as a consequence of U(VI) incorporation was also performed using solid-state ^29^Si magic angle spinning nuclear magnetic resonance (MAS-NMR) spectroscopy.

## Results and discussion

### U(VI) uptake by C–S–H solid phases

The reaction between U(VI)_(aq)_ and C–S–H phases was rapid and a yellow precipitate was instantaneously formed at all concentrations of U(VI) and all Ca/Si ratios (Supporting Information, Fig. 1). The uptake of U(VI) at t = 0 was > 99 % for concentrations of 0.5 mM to 25 mM uranyl nitrate, but at 50 mM the uptake decreased to ~ 60% at the same time point (Supporting Information, Fig. 2). The point of saturation for U(VI) uptake by C–S–H phases at a solid to liquid ratio of 25 g L^−1^ was achieved between 25 and 50 mM U(VI) and further analysis (Supporting Information, Fig. 2) determined that the point of U(VI) saturation was between 25 and 30 mM.

**Figure 1:**
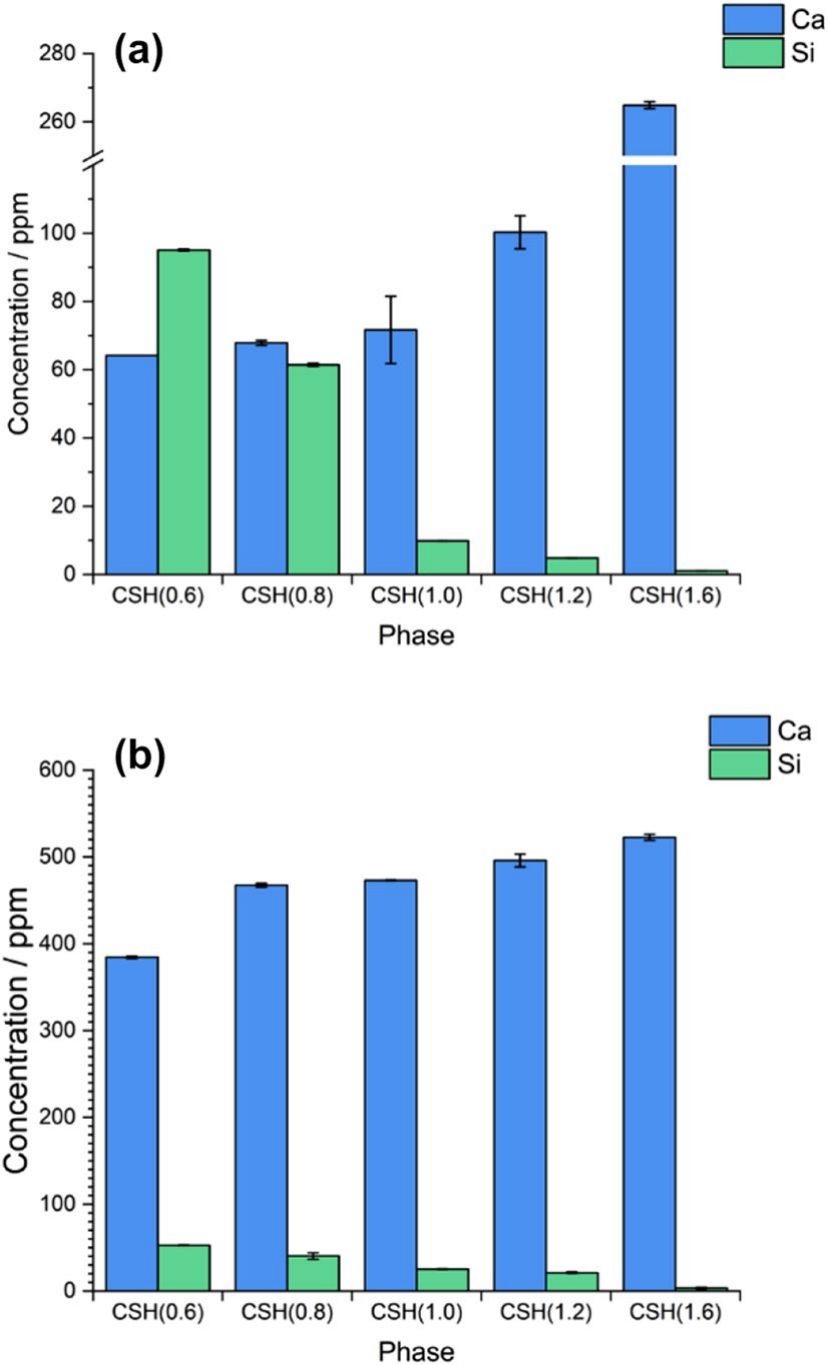
Aqueous concentrations of Ca and Si released to solution. For (**a**) 0.5 mM U(VI)-contacted and (**b**) 10 mM U(VI)-contacted C–S–H phases, with starting Ca/Si ratios ranging from 0.6 to 1.6, as labelled.

The pH measurements for the suspensions containing the C–S–H phases after contact with 0.5 mM (initial pH = 3.6 ± 0.1) and 10 mM (initial pH = 2.8 ± 0.0) U(VI) are given in Table 1. The pH values of the suspensions increased with increasing Ca/Si ratio, as a result of the release of Ca from the C–S–H phases driven by the acidic uranyl nitrate solution (Fig. 1).

**Table 1:** pH values of U(VI)-contacted C–S–H solutions after 48 h and final C–S–H composition after contact.

Initial C–S–H(X)	[U(VI)]/mM	pH	Estimated C–S–H(X)	Measured C–S–H(X)
Blank solution	0.5	3.6 ± 0.1		
CSH(0.6)		9.6 ± 0.0	CSH(0.60)	CSH(0.81)
CSH(0.8)		10.4 ± 0.0	CSH(0.80)	–
CSH(1.0)		11.5 ± 0.0	CSH(1.00)	–
CSH(1.2)		11.6 ± 0.0	CSH(1.19)	CSH(0.96)
CSH(1.6)		12.2 ± 0.0	CSH(1.58)	–
Blank solution	10	2.8 ± 0.0		
CSH(0.6)		9.2 ± 0.0	CSH(0.57)	CSH(0.83)
CSH(0.8)		9.3 ± 0.0	CSH(0.76)	–
CSH(1.0)		9.5 ± 0.0	CSH(0.96)	–
CSH(1.2)		10.0 ± 0.0	CSH(1.15)	CSH(0.93)
CSH(1.6)		11.2 ± 0.2	CSH(1.56)	–

Estimated and measured C–S–H compositions are derived from mass balance of solution concentrations present in Fig. 1 and from analysis of ^29^Si NMR data shown in Fig. 7, respectively. Errors on pH values represent one standard deviation of triplicate measurements.

At concentrations of 0.5 mM U(VI), the amount of Ca leached from C–S–H increased with the increasing Ca/Si ratio (Fig. 1a), while the amount of Si decreased, as expected from the lower amount of Si available in the system. The Si concentrations leached to solution were generally lower than those of Ca, except for the CSH(0.6) phase. At concentrations of 10 mM U(VI) (Fig. 1b), the amount of Ca leached was significantly higher than that of Si, with the amount of Ca leaching increasing with increasing Ca/Si ratio of the C–S–H phase. The amount of Si leached also decreased with increasing Ca/Si ratio, again attributed to the lower amount of Si available in the system. Table 1 shows the estimated Ca/Si ratio of each sample, determined from mass balance of the solution concentrations.

**Figure 2:**
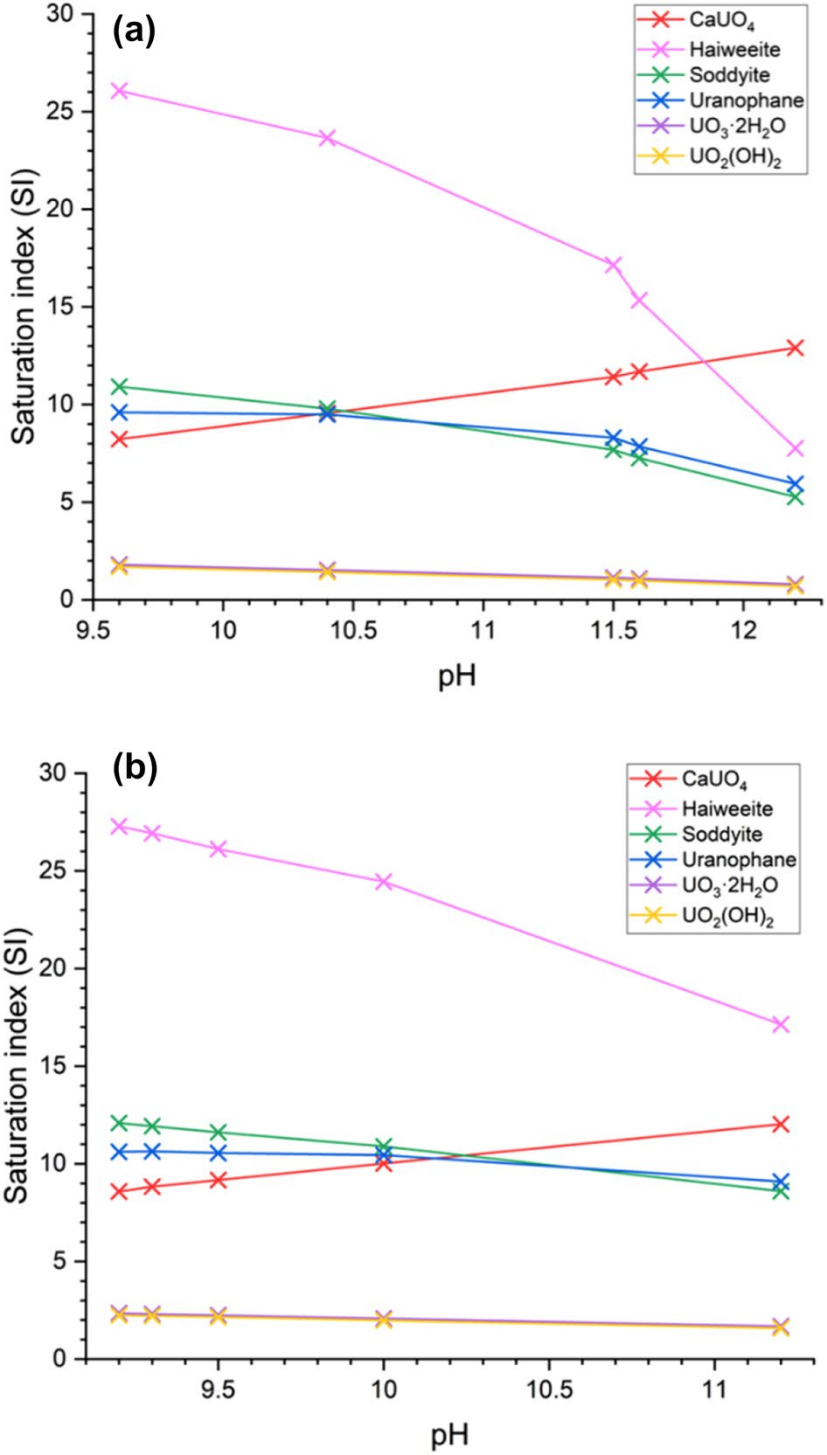
Saturation index estimations for U(VI)-containing phases. For (**a**) 0.5 mM U(VI)-contacted and (**b**) 10 mM U(VI)-contacted C–S–H systems at pH values associated with the starting Ca/Si ratio.

The results from geochemical modelling based on solution concentrations and pH measurements for the C–S–H systems contacted with 0.5 mM and 10 mM U(VI) are shown in Fig. 2. The type of U-containing phases identified as being saturated in the systems did not vary with U concentration, but included: calcium uranate (CaUO_4_); haiweeite (Ca(UO_2_)_2_(Si_5_O_12_)(OH)_2_⋅6H_2_O); soddyite ((UO_2_)_2_(SiO_4_)·2H_2_O); uranophane (Ca(UO_2_)_2_SiO_3_(OH)_2_·5H_2_O); metaschoepite (UO_3_·2H_2_O); and uranium hydroxide (UO_2_(OH)_2_). Although the calcium uranate phase identified by the model is a high temperature phase^17^, hydrous forms of calcium uranate are known to exist and are typically solubility limiting at high pH^18^.

### Phase analysis before and after U(VI) contact

**Figure 3:**
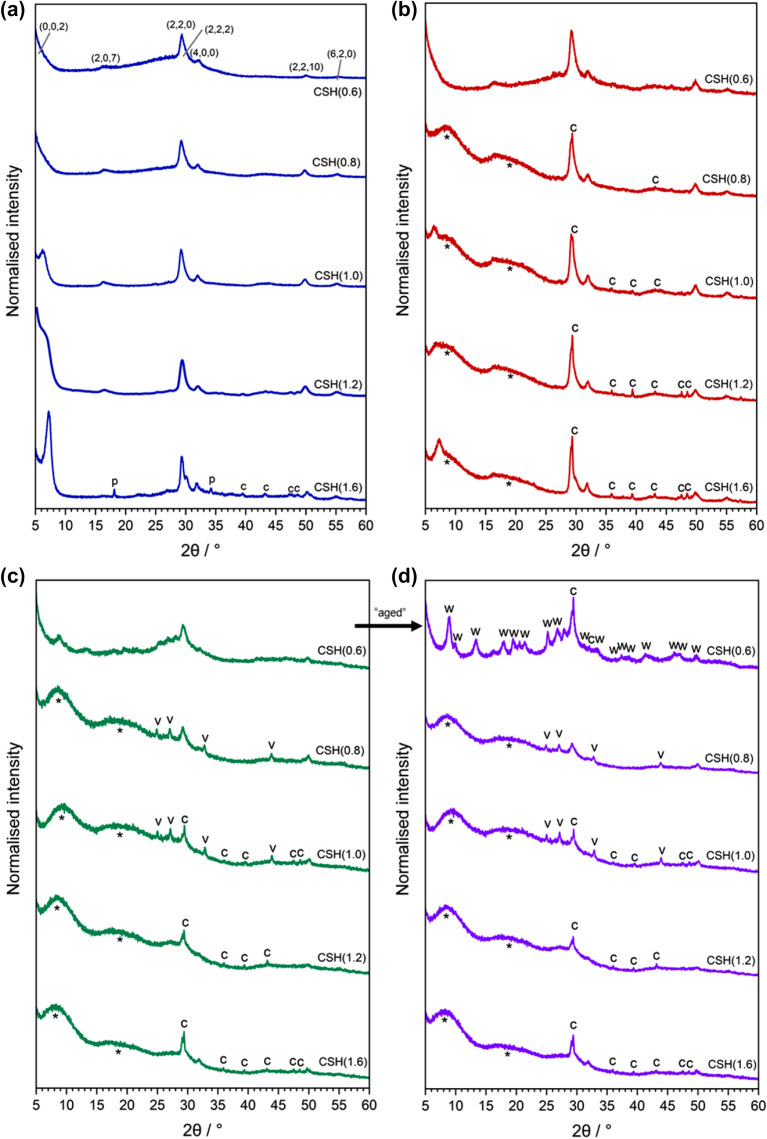
X-ray diffraction patterns of C–S–H phases. (**a**) before contact with U(VI) nitrate, indexed as C–S–H(I)/tobermorite (PDF card no 00–045-1480); (**b**) 0.5 mM U(VI)-contacted C–S–H phases; (**c**) 10 mM U(VI)-contacted C–S–H phases and; (**d**) 1 month aged 10 mM U(VI)-contacted C–S–H phases. Where c = calcite (CaCO_3_; PDF card no. 01-078-4614); p = portlandite (Ca(OH)_2_; PDF card no 04-006-9147); v = vaterite (PDF card no. 04-017-8634); w = “Ca-weeksite” (i.e. Ca_2_(UO_2_)_2_Si_6_O_15_·10H_2_O; PDF card no.00-012-0461/2).

X-ray diffraction patterns for the C–S–H phases before and after contact with 0.5 mM and 10 mM U(VI), are shown in Fig. 3. For the non-contacted C–S–H minerals (Fig. 3a), peaks for “C–S–H(I)” (tobermorite, Ca_5_Si_6_(O,OH)18·5H_2_O; PDF card no. 00-045-1480) were identified. Only in the CSH(1.6) phase was there evidence of portlandite (Ca(OH)_2_; PDF card no. 04-006-9147)^19^, which indicates that the maximum incorporation of Ca into the C–S–H phase using this particular synthesis method had been achieved^20^. Although every attempt was made to exclude CO_2_ during the synthesis, calcite (CaCO_3_; PDF card no. 01-078-4614) was observed for CSH(1.6), which was apparently more susceptible to carbonation than the other phases^21^. Similarly, the CSH(0.6) phase showed some increased areas of diffuse scattering/amorphicity in the diffraction pattern relative to the other C–S–H phases. This is indicative of an excess of silica (e.g. maximum incorporation of SiO_2_ into the C–S–H phase), reflective of the lower limit of Ca/Si ratios achievable^22^.

After contact with 0.5 mM U(VI), peaks for C–S–H(I) were maintained at all Ca/Si ratios of C–S–H (Fig. 3b); however, contact with 10 mM U(VI) appeared to result in a significant structural change in the C–S–H phases and only the main (2 2 0) reflection at ~ 29° 2θ and the (2 2 10) reflection at ~ 50° 2θ for C–S–H(I) were observed (Fig. 3c). There was also apparent increased amorphicity evidenced by increased diffuse scattering in the diffraction patterns, indicative of the formation of Ca-depleted Si-containing gel at the surface of the C–S–H phases.

Additional peaks were observed in the diffraction pattern of 10 mM U(VI)-contacted CSH(0.6), between 5 and 30° 2θ. These peaks were assigned to a “calcium-uranyl-silicate-hydrate phase”^23^ (PDF card no. 47-0497) with the chemical formula Ca_2_(UO_2_)_2_Si_6_O_15_·10H_2_O. Since this phase has a U:Si ratio of 1:3, which is the same as in the weeksite mineral group (with general chemical formula = K_2_(UO_2_)_2_(Si_5_O_12_)(OH)·4H_2_O)^24–26^, it is hereafter denoted as “Ca-weeksite”. A repeat measurement of this sample, more than one month after the U(VI)-contact experiment, showed an increase in the intensity of the diffraction peaks pertaining to the Ca-weeksite phase (Fig. 3d, denoted “w”), suggesting that this mineral phase became more crystalline with time.

For the remainder of the U(VI)-contacted C–S–H phases, there were no obvious additional peaks that could be attributed to U-containing phases, and repeat XRD measurements of the phases after one month did not reveal any further crystallisation. However, the loss of intensity of the C–S–H peaks and increased amorphous contribution to the signal after contact with 10 mM U(VI) for all other CSH(X) phases is indicative of the formation of disordered phases, likely Si-gel at the surface of the C–S–H minerals. Carbonation during analysis and/or drying was evident in all of the phases by the identification of peaks for calcite, and for the 10 mM U(VI)-contacted CSH(0.8) and CSH(1.0) phases only,

vaterite (CaCO_3_, PDF card no. 04-017-8634).

### Local U(VI) coordination environment

**Figure 4:**
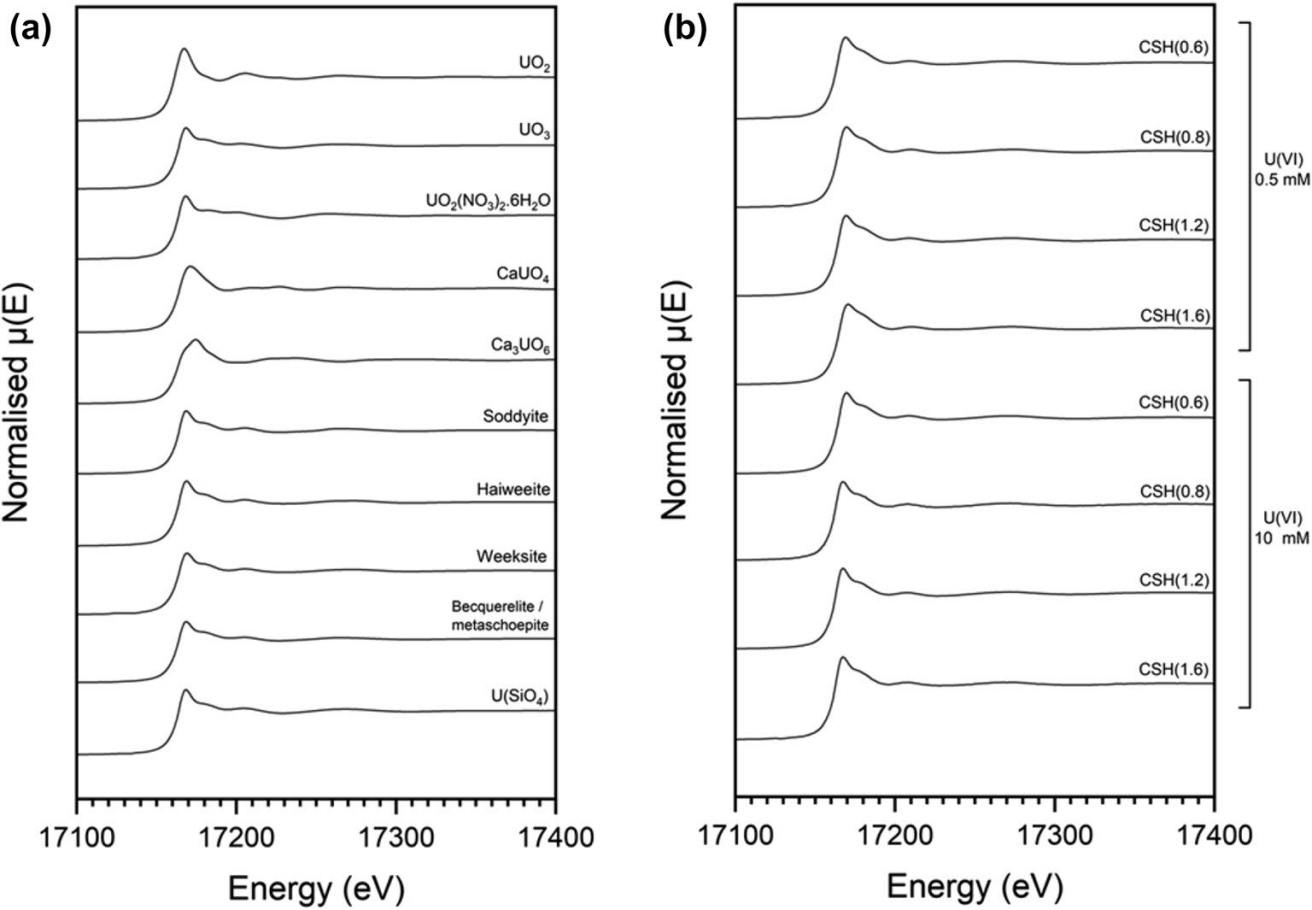
Uranium L_III_-edge X-ray absorption spectra. For (**a**) U ceramic and mineral standards and; (**b**) U(VI)-contacted C–S–H phases. Soddyite = (UO_2_)_2_SiO_4_·2H_2_O; haiweeite = Ca[(UO_2_)_2_Si_5_O_12_(OH)_2_]·3H_2_O; weeksite = K_2_(UO_2_)_2_Si_6_O_15_·4H_2_O; bequerelite = Ca(UO_2_)_6_O_4_(OH)_6_·8H_2_O; and metaschoepite = UO_3_·xH_2_O (x < 2). The latter two minerals were found to co-exist within the single standard utilised.

The X-ray absorption spectra obtained at the U L_III_-edge for the selected U(VI)-contacted C–S–H phases and U standards are shown in Fig. 4. The XANES spectra of the mineral uranyl silicates soddyite ((UO_2_)_2_SiO_4_·2H_2_O)), haiweeite (Ca[(UO_2_)_2_Si_5_O_12_(OH)_2_]·3H_2_O), and weeksite (K_2_(UO_2_)_2_Si_6_O_15_·4H_2_O), a mixed becquerelite (Ca(UO_2_)_6_O_4_(OH)_6_·8H_2_O)/metaschoepite (UO_3_·xH_2_O (x < 2) mineral, and coffinite (USiO_4_), given in Fig. 4a, show very similar spectral features and are not easily distinguishable. They are also very similar to that of the UO_3_ XANES spectrum. The spectra for CaUO_4_ and Ca_3_UO_6_, however, are more easily discernible from the former, and from one another. The spectra for the U(VI)-contacted C–S–H phases given in Fig. 4b do not appear to vary significantly between the two concentrations of U(VI).

The results from the geochemical modelling of the aqueous U(VI)/C–S–H systems in this work indicated that uranyl silicate minerals (e.g. haiweeite, soddyite, uranophane) or calcium uranate (e.g. CaUO_4_·xH_2_O) are likely to be the predominating solid phases under the experimental conditions adopted, along with a possible contribution from metaschoepite (UO_3_·2H_2_O) or other uranyl-oxy hydroxide phases (Fig. 2). The formation of uranyl silicate minerals has previously been documented for U(VI) in cementitious systems (e.g. uranophane and/or soddyite)^14,15^, in agreement with the observation in the present study of a Ca-weeksite-type phase (Ca_2_(UO_2_)_2_Si_6_O_15_·10H_2_O) for CSH(0.6) contacted with 10 mM U(VI). Becquerelite has been tentatively identified to form on U(VI)-contacted cementitious surfaces^27^ and, at higher concentrations of U(VI), the solubility of U(VI) is shown to be limited by the precipitation of calcium uranate phases^13,16^. Given these rationalisations, and the similarities and distinctions between the XANES spectra of the standards, the combination of standards allowed at any one time in the linear combination fitting of the U(VI)-CSH(X) samples was therefore limited to a maximum of 3 of the total, to allow for the potential fitting of the local coordination of: (1) a uranyl-silicate or uranium-silicate coordinated phase (e.g. soddyite, haiweeite, weeksite, coffinite) or a uranyl-oxy/hydroxide-type phase (e.g. UO_3_, mixed becquerelite/metaschoepite); (2) a calcium uranate-type phase (i.e. CaUO_4_); or (3) a tri-calcium uranate-type phase (i.e. Ca_3_UO_6_).

**Figure 5:**
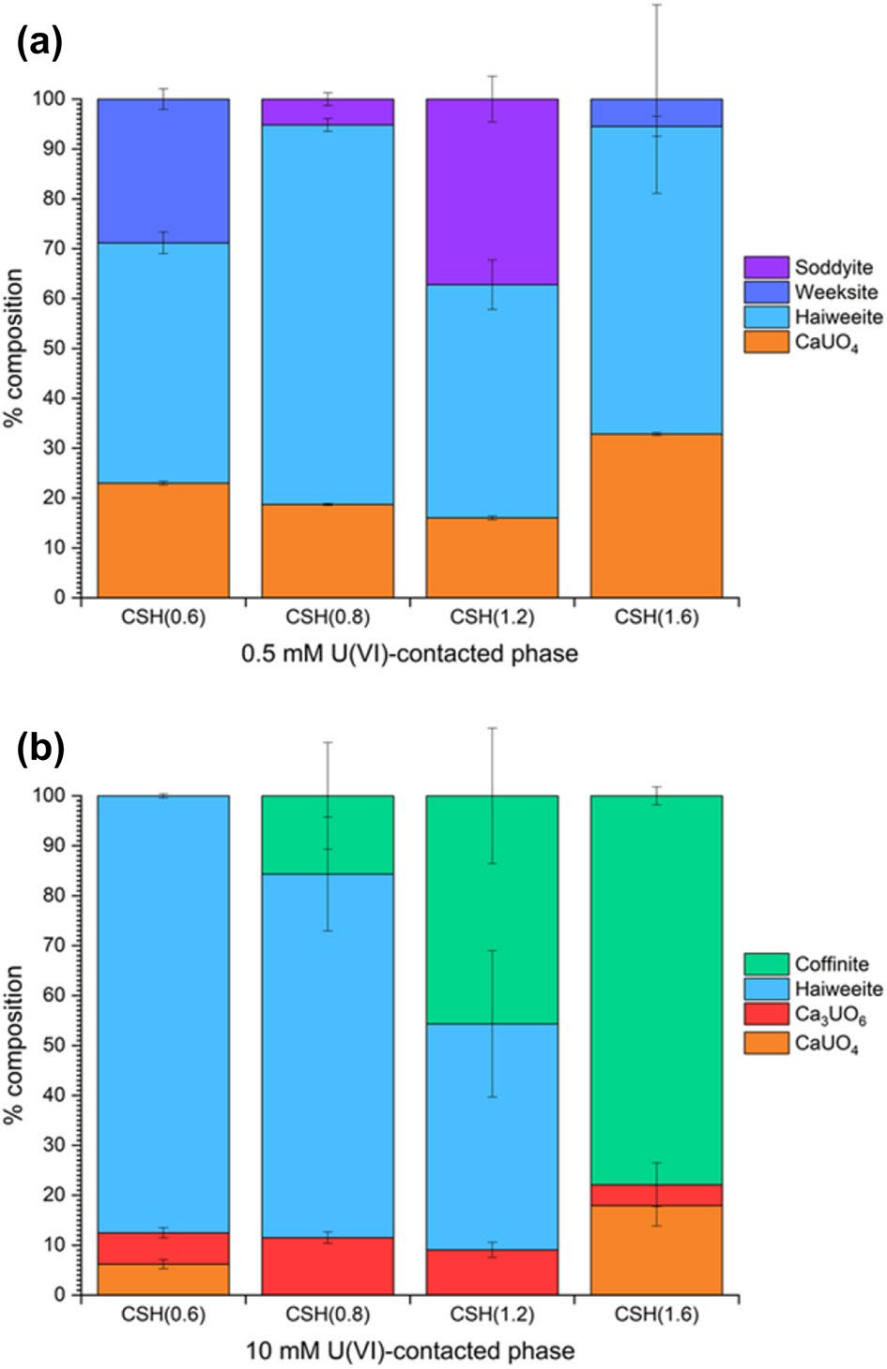
Linear combination fitting analysis of U L_III_-edge XANES. For (**a**) 0.5 mM and (**b**) 10 mM U(VI)-contacted C–S–H phases compared to the U standards.

Since XANES spectra of uranyl silicates are not easily distinguishable at the U L_III_-edge, the statistical significance of the specific assignments of individual uranyl silicate mineral phases was investigated. All fits were inspected to check that reasonable values and errors were achieved, after which the fit with the lowest R-factor was selected as representative. The results from the linear combination fitting are given in Fig. 5 and full details of the analysis are presented in Supplementary Information Table 2.

For the 0.5 mM U(VI)-contacted C–S–H minerals, the spectrum of CaUO_4_ was shown to contribute to the signal in all of the fits, at ~ 15–30%. The remainder of the signal was attributed to spectra of uranyl silicate phases including, in all cases, haiweeite. For the CSH(0.6) and CSH(1.6) phases, the inclusion of the weeksite spectrum was required to give the best fit and, for CSH(0.8) and CSH(1.2), a contribution to the spectra from soddyite improved the fit. For the 10 mM U(VI)-contacted C–S–H minerals, the linear combination fitting suggested that the spectra of haiweeite and coffinite contributed to the majority of the fit (> ~ 80%). The remainder was attributed to calcium uranate phases. A contribution from Ca_3_UO_6_ was fitted in all C–S–H phases; however, CaUO_4_ was only fitted for CSH(0.6) and CSH(1.6). It should be noted that coffinite is unlikely to represent the form of U in these samples, as it has an oxidation state of U(IV). Its inclusion in the linear combination fit, in which the positions of E_0_ are aligned, is simply reflective of the presence of U coordinated by Si atoms.

**Figure 6:**
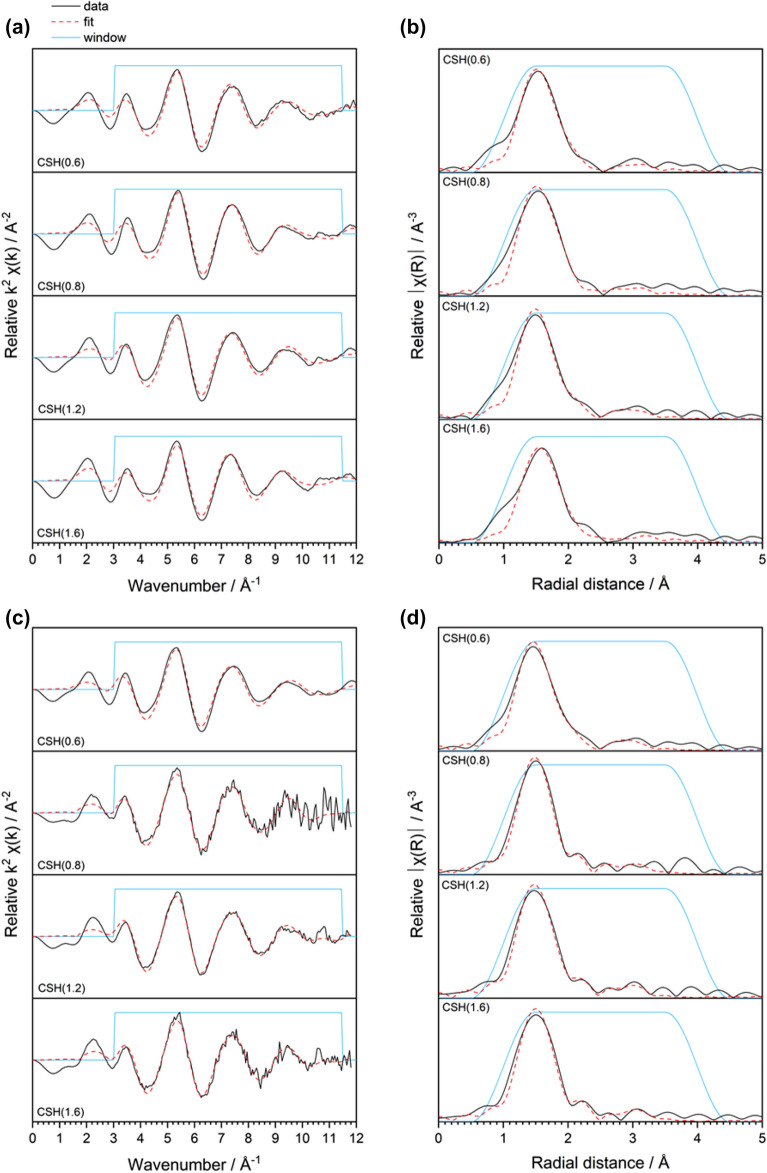
Local coordination analysis of U(VI) in contact with C–S–H phases. (**a**) k^2^-weighted spectra and (**b**) corresponding Fourier transformed radial plots for 0.5 mM U(VI)-contacted C–S–H phases; (**c**) k^2^-weighted spectra and (**d**) corresponding Fourier transformed radial plots for 10 mM U(VI)-contacted C–S–H phases.

To gain insight beyond the qualitative linear combination fitting, analysis of the U L_III_-edge EXAFS region was performed. The k^2^-weighted spectra and the Fourier transform radial distribution profiles of the U(VI)-contacted C–S–H minerals, and subsequent EXAFS model fits for each are shown in Fig. 6, with the fit parameters given in Table 2. k^2^-weighting was selected for plotting since the majority of signal contributions arose from nearest neighbour oxygens and because there was poor signal resolution at higher k values (due to low U content).

**Table 2:** EXAFS model parameters for U(VI)-contacted C–S–H minerals.

[U(VI)] (mM)	Phase	R-factor	ΔE/eV	Scatterer	R/Å	N	σ^2^
0.5	CSH(0.6)	0.015	8(2)	O_ax_^†^	1.829(9)	2	0.0026(5)
				O_eq1_	2.24(2)	3	“
				O_eq2_	2.39(3)	2	“
				Si	3.12(5)	1	0.012(8)
	CSH(0.8)	0.023	9(2)	O_ax_^†^	1.816(9)	2	0.0018(6)
				O_eq1_	2.26(2)	3	“
				O_eq2_	2.45(3)	2	“
				Si	3.08(5)	1	0.008(7)
	CSH(1.2)	0.021	8(2)	O_ax_^†^	1.814(10)	2	0.0019(6)
				O_eq1_	2.25(2)	3	“
				O_eq2_	2.42(3)	2	“
				Si	3.12(5)	1	0.008(6)
	CSH(1.6)	0.029	7(3)	O_ax_^†^	1.832(13)	2	0.0033(8)
				O_eq1_	2.23(2)	3	“
				O_eq2_	2.36(4)	2	“
				Si	3.13(6)	1	0.010(9)
10	CSH(0.6)	0.014	6(2)	O_ax_^†^	1.804(9)	2	0.0021(5)
				O_eq1_	2.25(2)	3	“
				O_eq2_	2.42(3)	2	“
				Si	3.13(5)	1	0.010(7)
	CSH(0.8)	0.023	9(2)	O_ax_^†^	1.816(9)	2	0.0018(6)
				O_eq1_	2.26(2)	3	“
				O_eq2_	2.45(3)	2	“
				Si	3.08(5)	1	0.008(7)
	CSH(1.2)	0.015	10(2)	O_ax_^†^	1.817(8)	2	0.0021(5)
				O_eq1_	2.28	3	“
				O_eq2_	2.48	2	“
				Si	3.14	1	0.010(7)
	CSH(1.6)	0.020	12(2)	O_ax_^†^	1.824(9)	2	0.0021(5)
				O_eq1_	2.28(2)	3	“
				O_eq2_	2.48(2)	2	“
				Si	3.14(6)	1	0.011(9)

R, effective interatomic distance; N, coordination number; σ^2^, Debye–Waller factor. Numbers with no errors have been fixed in the model.

^†^MS pathways also fitted at twice the distance of R_Oax_.

All of the U(VI)-contacted C–S–H phases could be fitted with the same model, regardless of U(VI) concentration or Ca/Si ratio. An O_ax_ shell was fitted with N_Oax_ = 2 at a distance of ~ 1.8 Å and a split O_eq_ shell was fitted with N_Oeq1_ = 3 and N_Oeq2_ = 2 at distances of ~ 2.2 Å and ~ 2.4 Å, respectively, in each case. A Si scatterer with N_Si_ = 1 was also fitted at a distance of ~ 3.1 Å in each model. These data are similar to those previously reported for C–S–H contacted with U(VI)^12,15^, as well as for the uranyl-silicate phases, uranophane^12^, soddyite^12^ and schoepite^28^.

Due to the low resolution of the data at higher k values, fitting beyond the Si shell was considered unreliable. Although a distance for Ca was generated in the FEFF calculation at distance of ~ 3.6 Å, the radial features did not appear well resolved beyond ~ 3.5 Å. For example, there appeared to be a prominent feature at ~ 3.9 Å in the 10 mM U(VI)-contacted CSH(0.8) phase; however, the data at high k values for this phase had the lowest signal to noise ratio of all the phases, so this feature was attributed to the low data resolution in this region. It is therefore concluded that, since Ca did not improve the fit, uranyl silicate phases dominated the EXAFS signal.

### Local coordination of Si

**Figure 7:**
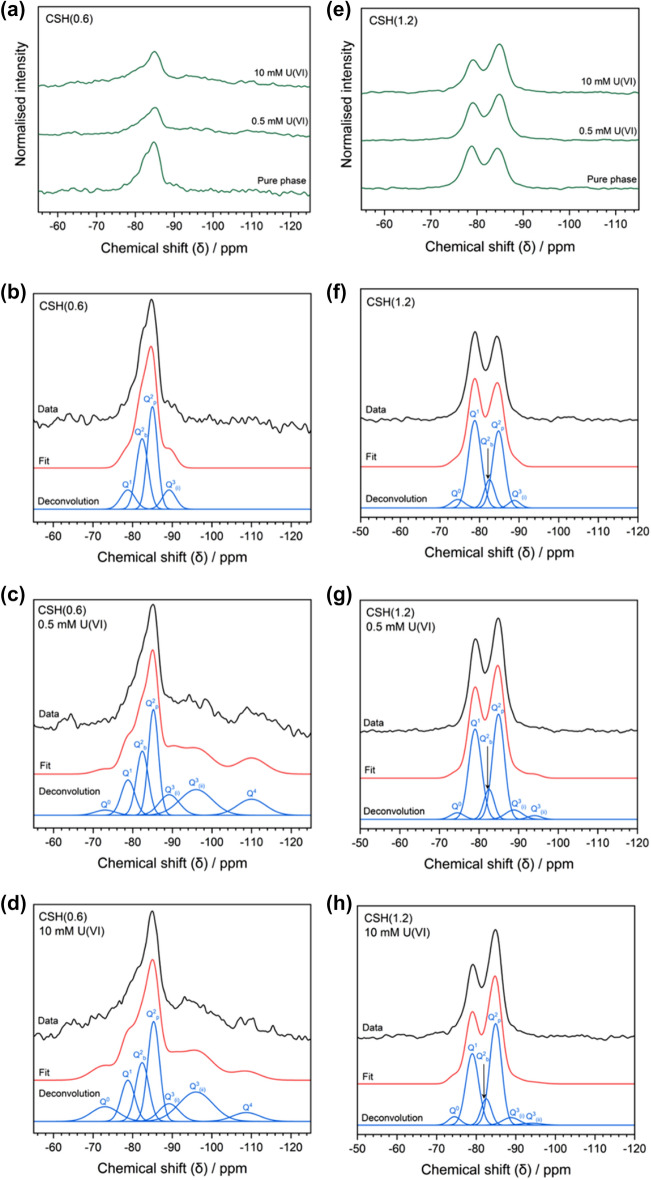
Normalised ^29^Si MAS-NMR spectra of CSH samples. (**a**) Normalised ^29^Si MAS-NMR spectra of CSH(0.6) under varying U(VI)-contacting conditions; (**b–d**) non-normalised ^29^Si spectra of CSH(0.6) under various U(VI)-contacting conditions with Si Q^n^ species deconvolution; (**e**) normalised ^29^Si spectra of CSH(1.2) under various U(VI)-contacting conditions and; (**f–h**) non-normalised ^29^Si spectra of CSH(1.2) under various U(VI)-contacting conditions with Si Q^n^ species deconvolution.

The normalised ^29^Si MAS-NMR spectra of the highest and lowest Ca/Si ratio C–S–H samples, CSH(0.6) and CSH(1.2), before and after U(VI)-contact are shown in Fig. 7. The non-contacted CSH(0.6) phase demonstrated a ^29^Si signal in the region of δ_obs_ = − 78 to − 90 ppm (Fig. 7a), while the corresponding CSH(1.2) phase displayed a signal between δ_obs_ = − 74 to -90 ppm, with two clearly resolved peaks. A slight shift in intensity to higher (less negative) δ_obs_ was observed. The individual assignments of different tetrahedral silicon Q^n^ species in the CSH(0.6) and CSH(1.2) series are also shown in Fig. 7 and the relative percentages of each Q^n^ species for each system are also given (Table 3), along with the NMR-derived Ca/Si ratios.

**Table 3:** Percentage of Si Q^n^ species in CSH(0.6) and U(VI)-contacted CSH(0.6) systems and CSH(1.2) and U(VI)-contacted CSH(1.2) systems, with the chemical shift (δ_obs_, ppm) given in brackets below each percentage value.

	Ca/Si	% contribution to signal (chemical shift/ppm)						
		Q^0^	Q^1^	Q^2^_b_	Q^2^_p_	Q^3^_(i)_	Q^3^_(ii)_	Q^4^
CSH(0.6) (noise ± 7.8%)	0.67	–	12 (− 78.8)	35 (− 82.5)	43 (− 85)	11 (− 89.2)	–	–
CSH(0.6) + U(VI) 0.5 mM (noise ± 7.9%)	0.81	3 (− 73)	12 (− 78.7)	18 (− 82.4)	26 (− 85.2)	10 (− 89.2)	20 (− 96)	11 (− 110)
CSH(0.6) + U(VI) 10 mM (noise ± 5.4%)	0.83	9 (− 73)	12 (− 78.8)	17 (− 82.4)	26 (− 85.3)	8 (− 89.2)	22 (− 96)	5 (− 108.7)
CSH(1.2) (noise ± 1.1%)	1.1	4 (− 74.5)	44 (− 78.8)	11 (− 82.5)	37 (− 84.8)	3 (− 88.8)		
CSH(1.2) + U(VI) 0.5 mM (noise ± 2.8%)	0.96	3 (− 74.5)	38 (− 79)	10 (− 82.5)	42 (− 84.9)	5 (− 88.8)	2 (− 94)	
CSH(1.2) + U(VI) 10 mM (noise ± 1.7%)	0.93	4 (− 74.5)	34 (− 79)	10 (− 82.5)	46 (− 84.9)	5 (− 88.8)		

The Ca/Si ratio was determined from ^29^Si NMR data, using Eq. (1).

The silicon Q^n^ species in the CSH(0.6) phase are typical of C–S–H at lower ratios of Ca/Si^29,30^. The Q^1^ contribution (~ 12%) is the signal arising from silicon tetrahedron dimers or silicon chain termination points, the Q^2^_b_ (~ 35%) and Q^2^_p_ (~ 43%) contributions arise from bridging and pairing silicon tetrahedron, respectively, and the Q^3^ contribution (~ 11%) is the signal arising from cross-linked silicon tetrahedra. The silicon Q^n^ species in the CSH(1.2) phase were also typical of a pure phase C–S–H at moderate range Ca/Si ratio^29–34^. The Q^1^ and Q^2^_p_ species contributed to the majority of the signal, at ~ 44% and ~ 37%, respectively. There was also a small Q^3^ signal identified (~ 3%) and, unlike in the CSH(0.6) phase, a small signal from Q^0^ species (hydrated silicon monomers; ~ 4%) from unreacted silica.

With addition of U(VI) to the CSH(0.6) phases, the Q^1^, Q^2^ and Q^3^ sites were maintained, albeit with a slight change in signal contribution, which signifies some retention of the original C–S–H structure. This is in good agreement with the XRD data that also displayed the presence of the C–S–H phases after contact with 0.5 mM and 10 mM U(VI); albeit to a lesser extent with 10 mM U(VI). The percentage contribution of the Q^1^ species remained at ~ 12% in both cases of U(VI) contact but there was also a small contribution from Q^0^ that was fitted at ~ 3% and ~ 9% for the 0.5 mM and 10 mM U(VI)-contacted phases, respectively, likely resulting from the dissolution of Ca from the solid phase, promoted by the low pH of the uranyl nitrate solution, leaving behind a disordered Si-rich gel at the surface. The relative signals from the bridging and pairing Q^2^ sites both decreased by a total of ~ 17% in each of the U(VI)-contacted samples, which is consistent with the calculated increase in Ca/Si ratio (Table 3). This somewhat unexpected result is postulated to be associated with mineral precipitation, as discussed later.

The percentage of the signal assigned to Q^3^ (i.e. Q^3^_(i)_) decreased on moving from pure phase CSH(0.6) to 0.5 mM U(VI)-contacted and 10 mM U(VI)-contacted phases. The emergence of an additional Gaussian contribution in the Q^3^ region at δ = − 96 ppm was also significant (denoted as Q^3^_(ii)_) at ~ 20% and ~ 22% for the lower and higher concentrations of U(VI), respectively. In addition to this, a Q^4^ signal (fully polymerised silicon tetrahedron) was also observed at δ =  ~ − 110 ppm with a contribution of ~ 11% and ~ 5% for these U(VI) concentrations, respectively. These assigned Q^n^ species indicate a change in the silicon environment as a result of U(VI) addition, with the potential formation of Q^3^ and Q^4^ species that may be attributed to formation of a uranyl silicate.

Contact of the CSH(1.2) phase with both U(VI) concentrations resulted in a decrease in the Q^1^ signal relative to an increase in the Q^2^_p_ signal, by ~ 5–6% at 0.5 mM U(VI) and an additional ~ 4% at 10 mM U(VI). This is a result of the higher release of Ca from the C–S–H phases, relative to silicon, which was further increased at the higher concentration of U(VI) (due to the lower pH), thus reducing the Ca/Si ratio of the C–S–H phase. The dominant presence of these Q^1^ and Q^2^ signals, however, indicates the retention of a C–S–H structure on addition of U(VI), albeit with a lower Ca/Si ratio (Table 3).

### Formation of uranyl silicates in low Ca/Si ratio C–S–H

It was expected that the Ca/Si ratio of CSH(0.6) would decrease upon contact with the low pH U(VI) nitrate solution, in accordance with the high release of Ca (Fig. 1). However, the Ca/Si ratio, calculated from the relative quantity of Q^1^ and Q^2^ Si species (Eq. 1), was shown to *increase* following contact with U(VI), from ~ 0.6 to ~ 0.8, for both the 0.5 mM and 10 mM U(VI) concentrations, respectively. This may be rationalised by considering that the formation of uranyl silicate phases—Q^4^ containing-species—require the liberation of Si from C–S–H to form on the outer surface of the C-S-H, which would result in an overall increase in the Ca/Si ratio of the C–S–H.

Indeed, a Ca-bearing uranyl silicate phase with formula Ca_2_(UO_2_)_2_Si_6_O_15_·10H_2_O (“Ca-weeksite”) was observed by XRD. Skakle et al*.*^23^ categorised this mineral in the uranyl silicate subgroup of weeksite (nominally K_2_(UO_2_)_2_(Si_5_O_13_)·4H_2_O), which has a U:Si ratio of 2:5 (or 2:6)^23,24,35–37^.

**Figure 8:**
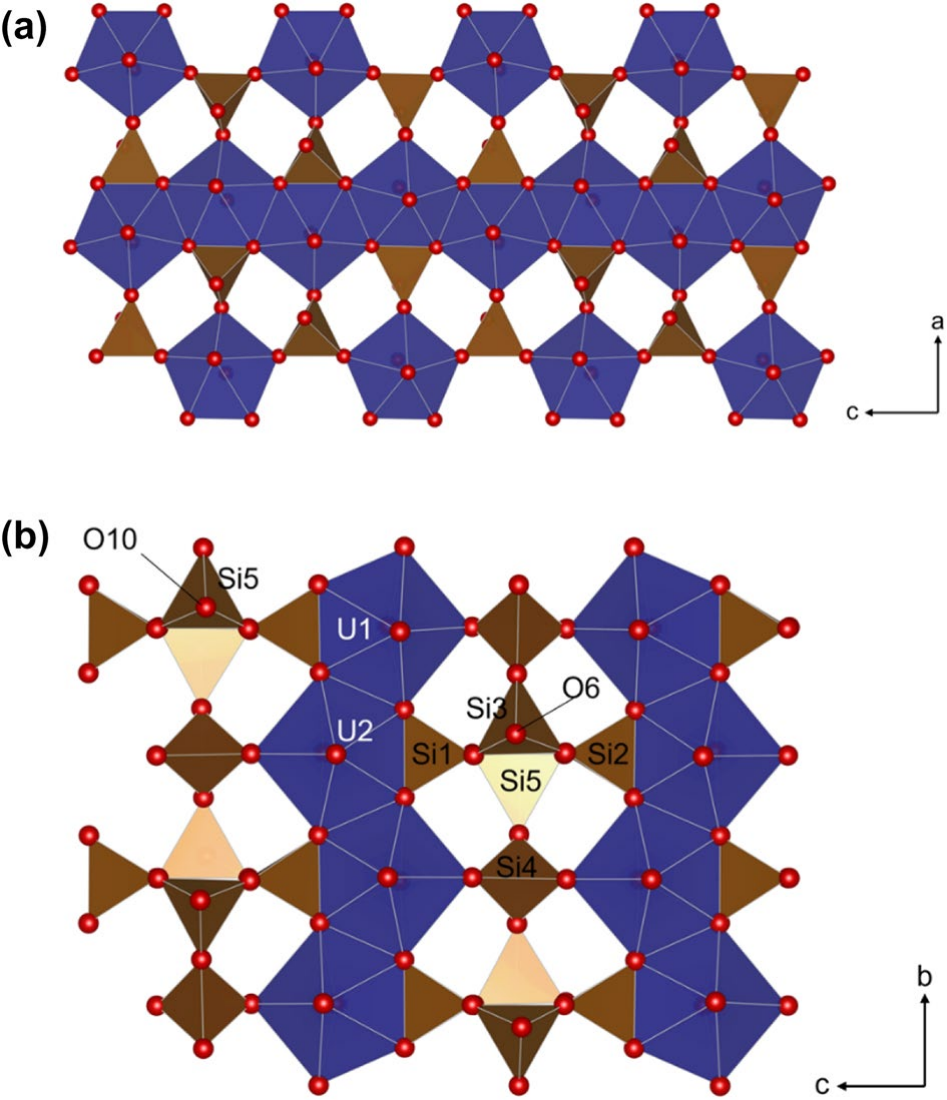
Crystal structures of uranophane-β (Ca(UO_2_)_2_SiO_3_(OH)_2_·5H_2_O) and haiweeite (Ca[(UO_2_)_2_Si_5_O_12_(OH)_2_]·3H_2_O). Silicon tetrahedron are in brown/beige and uranyl pentagonal bipyramids are in blue. (**a**) View of uranophane-β uranyl silicate layer along (0 1 0). (**b**) Uranyl silicate sheet in the crystal structure of haiweeite along (1 0 0). It should be noted that in weeksite (K_2_(UO_2_)_2_Si_6_O_15_·4H_2_O), the (1 0 0) view is the same as for haiweeite; however, the Si3 Q^3^ tetrahedron changes to a Q^4^ species whereby O6 connects to a subsequent Q^4^ species in the adjacent uranyl silicate sheet.

Further evidence for the presence of uranyl silicate phases is derived from the emergence of, and increase in, signals for Q^3^_(ii)_ and Q^4^ species observed in U(VI)-contacted CSH(0.6) with increasing U(VI) concentration. Uranyl silicate minerals tend to be sheet-structured with layers of uranyl silicates interspersed with interlayer cations and/or water molecules^24^. Uranophane (Ca(UO_2_)_2_SiO_3_(OH)_2_·5H_2_O), shown in Fig. 8a, exhibits only Q^3^(3U) species. Chains of pentagonal bipyramidal uranyl polyhedra make up uranyl chains, where the Q^3^(3U) species are edge-sharing with one pentagonal uranyl unit of one uranyl chain (2 × U) and vertex-sharing with another uranyl unit (1 × U) of the adjacent uranyl chain, to form uranyl-silicate sheets^38^. Weeksite (K_2_(UO_2_)_2_Si_6_O_15_·4H_2_O) exhibits Q^4^ and Q^4^(2U) species and demonstrates pentagonal bipyramidal uranyl chains, where the Q^4^(2U) species are edge sharing with one pentagonal uranyl unit (2 × U) to form the uranyl-silicate sheets. The sheets are connected by vertex-sharing Q^4^ species (connected to three Q^4^(2U) species and one further Q^4^ species). Haiweeite (Ca[(UO_2_)_2_Si_5_O_12_(OH)_2_]·3H_2_O), shown in Fig. 8b, exhibits Q^3^ and Q^4^(2U) species. The Q^4^(2U) species are also edge-sharing with pentagonal bipyramidal uranyl units (2 × U), as in weeksite. The Q^3^ species are then vertex-sharing with three of the Q^4^(2U) species, but unlike in weeksite (and as these are Q^3^ species), the sheets are not connected^25,39^.

Uranophane and weeksite could, therefore, account for the observations of singular Q^3^_(ii)_ and Q^4^ species, respectively. Given that the haiweeite structure exhibits both Q^3^ and Q^4^ species, this could account for the observed signals in the ^29^Si MAS-NMR spectra for the CSH(0.6) phases. The formula of the Ca_2_(UO_2_)_2_Si_6_O_15_·10H_2_O (Ca-weeksite) phase reported by Skakle et al*.*^23^ would also indicate that the silicon environment is not only attributed to Q^4^(*x*U) species since the ratio of oxygen atoms per silicon atoms, from the Si_6_O_15_ unit, is not equal to 4 (i.e. 15/6 ≠ 4). Skakle et al*.* categorised this phase in the weeksite sub-group, which also includes haiweeite. The amount of water (and OH groups) reported in the formula could vary and account for the existence of the silicon Q^3^_(ii)_ species observed by MAS-NMR.

### Formation of uranyl silicates in high Ca/Si ratio C–S–H

For the CSH(1.2) phase, the emergence and increase in the Q^3^_(ii)_ and Q^4^ signals in the ^29^Si MAS-NMR spectra after U(VI)-contact was less prominent. The Ca/Si ratio of the system was observed to decrease from ~ 1.1 to ~ 0.9 and a decrease in the ratio of Q^1^ to Q^2^ species was observed with increased addition of U(VI). This could be due to Ca release into solution, or from carbonation effects. The higher initial Ca/Si ratio resulted in better retention of the C–S–H phase during contact with low pH U(VI) solutions, which could account for why there was a less significant increase of the Q^3^_(ii)_ and Q^4^ signals (≤ 5%). This could be indicative of a U(VI) species that is physi-sorbed to the C–S–H surface, as discussed previously^12–16,40,41^, or the domination of calcium uranate species, whereby by the average Si signal by MAS-NMR would not be greatly perturbed.

Alternatively, the increase in signals for Q^3^_(ii)_ and Q^4^ species could arise from decalcification of the C–S–H structure due to calcium carbonate formation, independent of the presence of U(VI). This would lead to an increase in cross-linking of silicon tetrahedra and, therefore, an increase in Q^3^ species (and potentially Q^4^ species if complete decalcification and fully polymerised silicon sites were attained). However, a relative decrease in the Q^1^ to Q^2^ ratio and an associated decrease in the Ca/Si ratio of the C–S–H phase^41,42^ should also be observed. Given that the Ca/Si ratio was observed to increase in the CSH(0.6) system (from ~ 0.6 to ~ 0.8), it is concluded that the structural effect of C–S–H carbonation can be considered as negligible.

In addition to the formation of uranyl silicate phases, Macé et al*.*^12^ also reported the formation of a coexistent hydrous calcium uranate-type phase (CaUO_4_·*x*H_2_O) at U(VI) loadings of ~ 13 000 to ~ 45 000 ppm (mg kg^-1^) on C–S–H phases. Tits et al*.*^13^ also noted that at higher Ca/Si ratios of C–S–H, and increased U(VI) loadings, a calcium uranate phase was observed due to oversaturation. It is well understood that calcium uranate phases will be solubility limiting for U(VI) in cementitious waters with high Ca content^16,43,44^. Geochemical modelling identified the formation of calcium uranate in all of the U(VI)/C–S–H systems studied here and the results from XANES linear combination fittings were indicative of signal contributions from calcium uranate phases (CaUO_4_ or Ca_3_UO_6_).

In agreement with the current study, where Ca-weeksite phases appeared to become more crystalline over the period of 1 month, Sutton^43^ observed the same phenomenon for schoepite ((UO_2_)_8_O_2_(OH)_12_·12(H_2_O)) and hydrous calcium uranate phases. In the aforementioned study, solutions of uranyl nitrate were added to Ca(OH)_2_, combining to form mixed schoepite and calcium uranate precipitates, with an increasing calcium uranate content with increasing pH. The precipitate that formed as a result of this reaction was analysed by XRD and initially displayed diffuse scattering, but after ageing for 3, 6 and 9 weeks under anaerobic conditions (ambient temperature) an additional small, broad peak pertaining to either schoepite or calcium diuranate (CaU_2_O_7_) was observed. Sutton attributed this behaviour to the formation of a metastable state where the initial U(VI) phase formed is not the most thermodynamically stable but changes over time to a more stable phase. It was further postulated that such behaviour is the case for precipitates that form when solutions are oversaturated with respect to U(VI), and are therefore inherently disordered. This is in accordance with Ostwald’s step rule, which states that crystallisation from a solution will occur in a process such that thermodynamically unstable phases form first, followed by a thermodynamically stable step, or steps, and can be related to the process of “Ostwald ripening”^42,43^.

In summary, the results presented herein build on previous knowledge of uranyl silicate formation in these systems by considering other analogous minerals. While uranophane-like phases (Ca[(UO_2_)(SiO_3_OH)]_2_·5H_2_O) were postulated to form when U(VI) was contacted with low Ca/Si ratio C–S–H, in agreement with previous literature, at higher Ca/Si ratios, the formation of haiweeite (Ca(UO_2_)_2_(Si_5_O_12_)(OH)_2_⋅6H_2_O) and/or Ca-bearing weeksite (Ca(UO_2_)_2_(Si_5_O_12_)(OH)·4H_2_O) was evidenced. This is significant since the Ca/Si ratio of cement materials used in radioactive waste immobilisation varies considerably, depending upon the type material used as a supplementary additive. The effect of Ostwald ripening of the uranyl silicate phases over time has not previously been considered, and it is proposed that further research should focus on determining the evolving mineralogy of these U(VI)-bearing phases and the associated implications for the bulk cement matrix.

## Methods

### Materials

CaO of general-purpose grade (Fisher Scientific) and fumed AEROSIL 200 SiO_2_ were used for C–S–H synthesis. CaO was calcined at 900 °C for 10 h prior to use to eliminate any CO_2_ impurities or pre-hydration products. Ultra high-quality deionised water (referred to as UHQ hereafter) was used for all aqueous solutions and suspensions, generated by filtration to achieve a resistivity measurement of 18.18 MΩ cm. All weighing of precursors was performed under ambient conditions on the benchtop, but mixing, filtration and storage was carried out under an N_2_ atmosphere to minimise carbonation.

### C–S–H synthesis

C–S–H phases with Ca/Si ratios of 0.6, 0.8, 1.0, 1.2 and 1.6 (referred to as CSH(X), where X = Ca/Si ratio), were prepared. These ratios were selected to encompass C–S–H from the very lower limits of formation (e.g. due to high replacement with siliceous SCMs or Ca-depleted/aged cements in a GDF) and the very upper limits of formation (e.g. due to high replacement with lime-based powders or more Ca-rich/younger cements in a GDF). Stoichiometric amounts of CaO and SiO_2_ were weighed to achieve the desired theoretical Ca/Si ratios. The weighed powders were added to Ar-degassed UHQ at a solids-to-liquid (S/L) mass ratio of 15 g L^−1^. The resulting suspensions were mixed for a minimum of 7 days at 40 rpm on a rotary shaker under a N_2_ atmosphere, before being filtered gravitationally through a Whatman-542-ashless filter paper. The solids were left to dry at ambient temperature for ~ 1 week, before grinding into a fine powder for characterisation and were thereafter stored under N_2_. Solutions of the reaction mixtures were also removed and acidified for inductively coupled plasma—optical emission spectroscopy (ICP-OES) analysis (using a ThermoFisher iCAP Duo6300 instrument) to determine that the desired Ca/Si ratio had been achieved.

### U(VI)-contact experiments

C–S–H phases were contacted with 0.5, 5, 10, 25 and 50 mM uranyl nitrate (UO_2_(NO_3_)_2 (aq)_) (i.e. [U]_t=0_) solutions at a S/L mass ratio of 25 g L^−1^, and the suspensions were mixed at 40 rpm on a rotary shaker for 48 h, with sampling of the supernatant at 2, 4, 6, 12, 24 and 48 h. The results from this experiment concluded that U(VI) precipitation with the C–S–H phases was instant. The C–S–H phases contacted with U(VI) at concentrations of 0.5 mM and 10 mM for 48 h were then selected for further analysis, to represent “borderline trace” and “elevated” concentrations, respectively.

For aqueous elemental analysis, the solutions were filtered through 0.22 μm cellulose filters. The pH values were then measured before the solutions were acidified and prepared for ICP-OES analysis to measure U, Ca and Si concentrations. For solid state analyses, the resulting solids were dried under N_2_ at ambient temperature for ~ 1 week before being ground into a fine powder for characterisation, and subsequently stored under N_2_.

Geochemical modelling was performed using the Phreeqc Interactive 3.4.0-12927 software and the Lawrence Livermore National Laboratory (LLNL) thermodynamic database, to estimate the saturation index of mineral phases likely to form in aqueous solution under the experimental conditions of the U(VI)-contact studies. The results from ICP-OES analyses and the solution pH values were used for the model input for Ca and Si, and the U(VI) concentration was input as 0.5 mM or 10 mM.

### Solid state analysis

X-ray diffraction (XRD) measurements of all C–S–H phases were performed before and after U(VI)-contact experiments, using a Bruker D2 Desktop instrument. Powders were compressed into a 10 mm diameter recess on a low background Si(111) plate in a PMMA holder. For U-containing samples the compressed powder was covered with an acetate film held in place with a small amount of PVA adhesive, in accordance with alpha-powder handling protocols. Measurements were taken between 5 and 60° 2θ with a counting time of 1 s per step, in increments of 0.02° 2θ, using a 1 mm divergence slit.

Selected U(VI)-contacted C–S–H phases and a suite of U standards considered relevant to U(VI) secondary phase formation in Ca-rich and Ca-depleted cements (Supplementary Material, Table 1), were analysed by U L_III_-edge (17,166 eV) X-ray Absorption Spectroscopy (XAS) at beamline B18 at Diamond Light Source and beamline BMM (6-BM) at NSLS-II to obtain information in the XANES (X-ray absorption near edge spectroscopy) and EXAFS (Extended X-ray absorption fine structure) regions. The amount of material required to allow for transmission measurement at 1 absorption length was approximated using the Hephaestus programme^45^; for U(VI)-contacted C–S–H phases this was estimated based on the general chemical formula of the mineral phases and an assumption of 100% U(VI)_(aq)_ uptake from solution. The accurately weighed powders were pressed into pellets using a polyethylene-glycol (PEG) binder to enhance mechanical stability, pressed at ~ 1 tonne for ~ 1 min.

Both beamlines were equipped with a Si(111) monochromator: for B18, beam collimation was achieved using a Cr and Pt coated Si mirror, while for BMM a Rh coated Pt mirror was used^46^. An Y foil was used in the reference channel for monochromator calibration in both cases. The Athena programme was used for post-processing and normalisation of data^45^. Data calibration was performed by assigning the first inflection point of the derivative energy spectrum (i.e. E_0_) for a Y foil in the reference channel (Y K-edge, 17,038 eV). The value of E_0_ for each data set was then assigned to the position of the maximum inflection point of its derivative energy spectrum.

Linear combination fitting analysis was applied to the XANES region of the U L_III_-edge spectra using the Athena software. A combination of any four of the considered phases (see Supplementary Information Table 1) were allowed to be fit within the region of − 20 and + 30 eV from the position of E_0_. The value of ΔE for each phase fitted was recorded. The “best fit” for each sample was chosen based on a combination of prior knowledge of the system deduced from experimental data and geochemical modelling estimations, in addition to R-factor and χ^2^ values.

The Artemis programme was used for the generation of scattering pathways and fitting of models for the EXAFS region of the U(VI)-contacted C–S–H phases^45^. In Athena, prior to this, the fitting window for the Fourier Transform of k-space into R-space was selected between k = 3 and k = 11.5, using a Hanning window (dk = 0), before being imported into Artemis. Given evidence from the previous literature, it was reasonable to assume a uranyl silicate model for fitting the EXAFS region. Scattering paths were generated using FEFF calculations of the CIF file for β-uranophane (ICSD #250001)^38^, so that pathways for the next nearest O_ax_, O_eq_, Si (or Ca) neighbours could be generated and allowed to refine in the model. Pathways were fitted between ~ 1 and ~ 5 Å in R-space using a Hanning window (dR = 0). ΔE was allowed to vary as a global parameter. As well as single scattering (SS) pathways, multiple scattering (MS) pathways were considered for U-O_ax_-O_ax_ (linear) interactions. The value of the amplitude reduction factor (S_0_^2^) for a U absorber was determined previously in the fitted EXAFS model for UO_2_ as 0.9, using pathways generated from the CIF file for UO_2_ (ICSD #160814)^47^, and was thereafter fixed in the model for the fitting of all other phases.

Samples of CSH(0.6) and CSH(1.2) were also selected for analysis by ^29^Si solid-state MAS-NMR spectroscopy, both before and after contact with 0.5 mM and 10 mM U(VI), respectively. Powders were packed into 4 mm ZrO_2_ sample rotors and spectra were collected using a Bruker Avance III HD 500 spectrometer at 11.4 T, with a resulting Larmor frequency of 99.35 MHz for ^29^Si. ^29^Si chemical shifts were referenced to neat tetramethylsilane (TMS). A MAS rate of 12.5 kHz was applied. Conventional single pulse experiments were carried out using an optimised pulse length of 1.4 µs and a recycle delay of 45 s. 256 scans were acquired for each sample. Post-processing of the data was carried out using the TopSpin 4.0.6 software, and data were normalised by integrated area. Deconvolution of the spectra was performed by fitting Gaussian peaks to the total signal to determine the contribution from individual Q^n^ Si species. The Ca/Si ratio of the C–S–H phases and U(VI)-contacted C–S–H systems was calculated^48^ by using the Gaussian signals determined for Q^1^ and Q^2^ and applying them to:

1 $${\raise0.7ex\hbox{${{\text{Ca}}}$} \!\mathord{\left/ {\vphantom {{{\text{Ca}}} {{\text{Si}}}}}\right.\kern-0pt} \!\lower0.7ex\hbox{${{\text{Si}}}$}} = \frac{{\frac{3}{2}{\text{Q}}^{1} + \frac{2}{3}{\text{Q}}^{2} }}{{{\text{Q}}^{1} + {\text{Q}}^{2} }}$$

## Supplementary Information

Supplementary Information.

## Data Availability

The data that support the findings of this study are available from the corresponding author upon request.
