# Supplementary material for: Spectroscopic identification of Ca-bearing uranyl silicates formed in C–S–H systems
Source: Sci Rep. 2023 Feb 28;13:3374. doi: 10.1038/s41598-023-30024-0 (PMC9974962; doi:10.1038/s41598-023-30024-0)
Supplement: Supplementary file 1 — Supplementary Information. [file 41598_2023_30024_MOESM1_ESM.docx]

# Spectroscopic identification of Ca-bearing uranyl silicates formed in C-S-H systems: Supplementary Information

Antonia S. Yorkshire, Martin C. Stennett, Brant Walkley, John L. Provis, Luke T. Townsend, Latham Haigh, Neil C. Hyatt, Lucy M. Mottram and Claire L. Corkhill^*^

*corresponding author: c.corkhill@sheffield.ac.uk


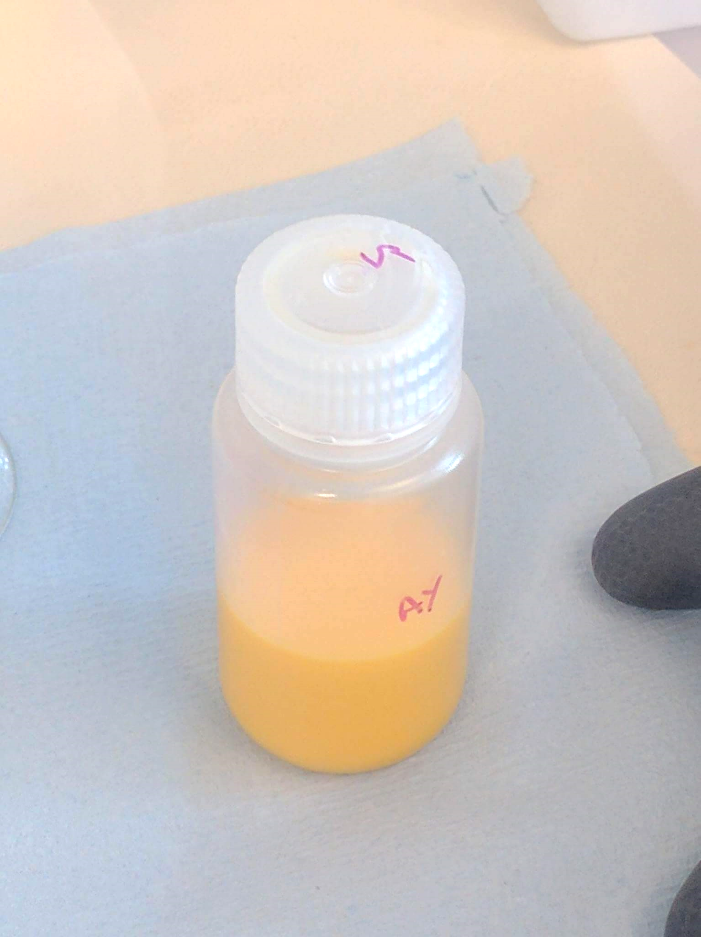


**Supplementary Information, Figure 1.** Yellow precipitate formed instantaneously upon contact of C-S-H with U(VI)-nitrate solution.


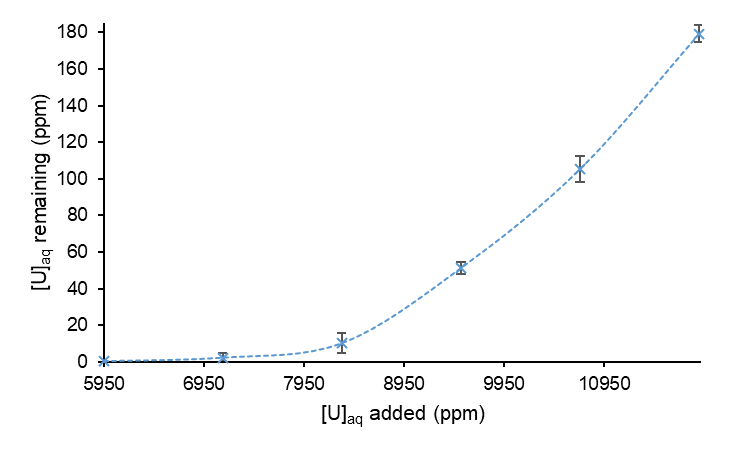


**Supplementary Information, Figure 2.** Graph showing the increase in [U] remaining in solution with the increase in concentration of U(VI) initially added to solution for U(VI)-CSH(0.6) contact experiments. Above ~5950 ppm (mg L^-1^) (25 mM) of U(VI) added the concentration of U(VI) remaining in solution reaches >0 ppm (mg L^-1^).

**Supplementary Information, Table 1**. List of U-bearing ceramic and mineral phase standards measured by U L_III_-edge XAS.

| **Standard (general formula)** | **Synthetic (S) or**  **natural (N)** | **Approximate U oxidation state** |
| --- | --- | --- |
| Uranium dioxide (UO_2_)^a^ | S | 4+ |
| Uranium trioxide (UO_3_) | S | 6+ |
| Uranyl nitrate (UO_2_(NO_3_)_2_·6H_2_O) | S | 6+ |
| Calcium uranate (CaUO_4_) | S | 6+ |
| Tricalcium uranate (Ca_3_UO_6_) | S | 6+ |
| Soddyite ((UO_2_)_2_SiO_4_·2H_2_O) | N | 6+ |
| Haiweeite (Ca[(UO_2_)_2_Si_5_O_12_(OH)_2_]·3H_2_O) | N | 6+ |
| Weeksite (K_2_(UO_2_)_2_Si_6_O_15_·4H_2_O) | N | 6+ |
| Becquerelite (Ca(UO_2_)_6_O_4_(OH)_6_·8H_2_O) / Metaschoepite (UO_3_·xH_2_O (x<2))^b^ | N | 6+ |
| Coffinite U(SiO_4_) | S | 4+ |

^a^UO_2_ was used energy for alignment and determination of S_0_^2^.

^b^This standard was determined to be a mixture of two phases upon XRD analysis. Both are stated.

**Supplementary Information, Table 2.** Weighted fraction of components in U L_III_-edge XANES signal of U(VI)-contacted C-S-H phases using Linear Combination Fitting (LCF). The energy shift in the first derivative of the absorption edge (ΔE), in eV, of the C-S-H spectrum, from that of the standard, is also shown.

|  | |  |  | **Uranyl / uranium silicate** | | | | | | | | **Uranate** | | | |
| --- | --- | --- | --- | --- | --- | --- | --- | --- | --- | --- | --- | --- | --- | --- | --- |
|  | |  |  | **Haiweeite** | | **Weeksite** | | **Soddyite** | | **Coffinite** | | **CaUO_4_** | | **Ca_3_UO_6_** | |
| **[U]** | **CSH(X)** | **R-factor** | **Weight** | **Fraction** | **ΔE** | **Fraction** | **ΔE** | **Fraction** | **ΔE** | **Fraction** | **ΔE** | **Fraction** | **ΔE** | **Fraction** | **ΔE** |
| **0.5** | **CSH(0.6)** | 3.6 x 10^-5^ | 1.013 | 0.488(22) | -0.511(89) | 0.292(21) | -0.329(187) |  |  |  |  | 0.233(4) | 1.081(71) |  |  |
|  | **CSH(0.8)** | 1.0 x 10^-5^ | 1.004 | 0.764(13) | 0.171(31) |  |  | 0.052(13) | -2.784(395) |  |  | 0.188(2) | 3.207(47) |  |  |
|  | **CSH(1.2)** | 9.6 x 10^-6^ | 1.006 | 0.470(50) | -0.268(42) |  |  | 0.374(46) | 0.512(86) |  |  | 0.161(4) | 3.059(109) |  |  |
|  | **CSH(1.6)** | 1.2 x 10^-5^ | 1.005 | 0.620(20) | 0.687(35) | 0.055(19) | -2.101(651) |  |  |  |  | 0.330(3) | 2.962(32) |  |  |
| **10** | **CSH(0.6)** | 1.8 x 10^-5^ | 1.001 | 0.876(4) | 0.529(14) |  |  |  |  |  |  | 0.062(9) | 4.127(315) | 0.063(10) | 3.151(315) |
|  | **CSH(0.8)** | 1.5 x 10^-4^ | 0.964 | 0.702(110) | -1.371(268) |  |  |  |  | 0.151(103) | -1.106(1.188) |  |  | 0.111(11) | 0.650(366) |
|  | **CSH(1.2)** | 2.2 x 10^-4^ | 0.981 | 0.444(144) | -1.218(723) |  |  |  |  | 0.448(133) | -1.024(683) |  |  | 0.089(15) | 0.498(548) |
|  | **CSH(1.6)** | 2.2 x10^-4^ | 1.004 |  |  |  |  |  |  | 0.782(18) | -1.195(93) | 0.180(41) | -0.800(730) | 0.042(44) | 0.754(2.149) |
